# Isolated Gastrointestinal Metastasis of Breast Carcinoma: A Case Report

**DOI:** 10.1155/2010/615923

**Published:** 2010-06-16

**Authors:** M. A. Titi, A. Anabtawi, A. D. Newland

**Affiliations:** ^1^Department of Surgery, Crosshouse Hospital, Kilmarnock, Scotland KA2 0BE, UK; ^2^Unity Hospital, 1555 Long Pond Road, Rochester, NY 14626, USA

## Abstract

*Purpose*. Gastrointestinal tract is one of the rare locations for breast cancer metastasis. This paper shows such metastasis may occur even in the absence of breast metastasis in other more common locations. *Case Report*. A 64-year old female was admitted to the hospital with abdominal discomfort and diarrhea. She had breast carcinoma treated 7 years previously with normal follow-up since. Colonoscopy showed hepatic flexure thickening that was confirmed to be breast metastasis. Staging investigations showed upper and lower gastrointestinal tract metastasis with negative findings elsewhere. *Conclusion*. Although more common causes for gastrointestinal symptoms should be excluded, however, a high index of suspicion of metastatic breast cancer is needed when such patients develop gastrointestinal symptoms.

## 1. Introduction

Breast carcinoma is the most common malignancy in women and is a leading cause for cancer-related mortality [1]. Invasive ductal carcinoma is the most common histological subtype (70%–80%), followed by invasive lobular carcinoma (10%–14%) [1, 2]. Approximately 50% of the patients with breast cancer will develop distant metastases during their lifetime. However, autopsy data reports even a higher incidence of subclinical distant metastases [3, 4]. The most common metastatic sites are lymph nodes, bones, lungs, and the liver [1].

Gastrointestinal (GI) tract is rarely reported to be involved by breast cancer metastasis. Nevertheless, breast cancer comes only second to melanoma in the rate of metastatic spread to the GI tract [5]. The true rate of this metastasis is believed to be underestimated as patients may be asymptomatic or present with nonspecific symptoms of nausea, vomiting, or diarrhea that can be easily missed or attributed to other etiologies like chemotherapy side effects. Metastasis to the colon is less commonly reported compared to gastric metastasis [6]. 

This case report highlights the importance of considering GI metastasis as part of the differential diagnosis when breast cancer patients present with persistent GI symptoms even in the absence of metastasis elsewhere.

## 2. Case Report

A 64-year old female was admitted to the hospital with symptoms of abdominal discomfort, diarrhea, and weight loss. The patient also had intermittent nausea and vomiting for few months treated as gastritis. Her past medical history included breast carcinoma which was diagnosed seven years earlier on screening mammography and was treated with lumpectomy and axillary node clearance followed by adjuvant radiotherapy and hormonal treatment (Tamoxifen) for five years. This was a 32 mm grade one infiltrating lobular carcinoma (T2 N0 M0) and hormone receptor (Estrogen (ER) positive, progesterone (PR) negative). Patient remained well with no clinical or radiological evidence of recurrence on her regular oncology follow-up visits. Patient was not on any current medications at this time of admission. She is nonsmoker. No family history of breast or gastrointestinal carcinomas.

Physical examination was remarkable for pale sclera, mild lower abdominal tenderness with no rebound tenderness and bowel sounds were present. Rectal exam was normal but fecal occult blood was positive. 

**Figure 1:**
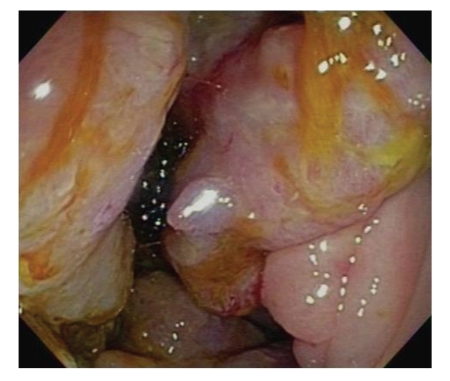
Large nodular hepatic flexure lesions as seen on Colonoscopy.

**Figure 2:**
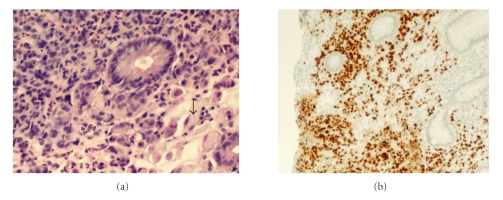
(a) Histopathology of biopsies taken from gastric lesions showing infiltrating adenocarcinoma with signet ring cell morphology (Arrow). (b) Immunohistochemistry of (a) showing positive ER.

Investigations showed normocytic normochromic anemia (Hemoglobin 9.4 grams per deciliter, Mean corpuscular volume 88.0 femtoliters, and Mean cell hemoglobin 29 picograms). White blood cell count was 11, 000 × 10^9^/l. Upper and lower endoscopy were performed which revealed multiple areas of nodular thickening involving the gastric antrum proximal duodenum and the hepatic flexure of the colon (Figure 1). Biopsies showed infiltrating adenocarcinoma with signet ring cell morphology suspicious for primary gastric tumor (Figure 2(a)). However, Immunoperoxidase stain of the specimen revealed cytokeratin (CK) 7 and ER both were positive (Figure 2(b)) while CK-20 and CDX2 were negative suggestive of metastatic breast carcinoma. PR and HER2 were negative. Staging investigations showed extensive metastasis to the upper and lower GI tract. No pulmonary or hepatic lesions were identified and nuclear bone scan was normal. 

The patient was managed initially with palliative hormonal treatment (Letrozole) which was changed to palliative chemotherapy due to disease progression. However, patient did not tolerate the side effects of chemotherapy and it was stopped. After seven months of her diagnosis with the breast cancer metastasis to the GI tract; the patient level of care was changed to comfort care and symptomatic treatment only and was transferred to inpatient hospice.

## 3. Discussion

The reported patient had extensive breast cancer metastasis to the upper and lower GI tract in the absence of any evident metastasis else where. Most patients in previous reports had the GI metastasis as part of a widely metastatic disease involving the commonly involved organs like the liver, lungs, or bones [7–9]. These findings advocate for keeping a high index of suspicion and a low threshold to further investigate persistent GI symptoms in patients with breast cancer even those with clinically early stage with no evident metastasis.

Upper GI tract specially the stomach has been more commonly reported to be involved with breast cancer metastasis than the colon and the lower GI tract [6–9]. An autopsy study of 707 breast cancer cases estimated the metastasis rate to the upper GI tract to be 6%–18% while the lower GI tract was involved in 8%–12% of the cases [6]. These autopsy results suggest that a significant number of breast cancer patients may have metastasis to the GI tract that are clinically underdiagnosed due to either being totally asymptomatic or causing nonspecific symptoms that could easily be missed. Indeed the patient in this report had nausea and vomiting prior to presentation which were considered secondary to gastritis. 

Histological variants of breast cancer have been shown to have different patterns of metastasis. Infiltrating lobular carcinoma was found to be responsible for the majority of metastasis to the GI tract [1, 9]. Nonetheless, some reports described GI metastasis of infiltrating ductal carcinoma [5]. The spread to the GI tract is believed to be blood borne in origin [10]. Differentiation of breast metastasis from other GI primaries can be difficult to establish using the histopathological appearance as in the reported case. Immunohistochemistry is crucial to establish the accurate diagnosis. Commonly used markers include ER and PR receptors, CK 7, CK 20, and gross cystic disease fluid protein 15 (GCDFP-15) [11, 12]. 

Management of breast cancer metastasis to the GI tract commonly involves systemic hormonal or chemotherapy treatment. The surgical role is usually palliative and is mainly indicated in cases of symptomatic stenosis or complete obstruction. The reported overall response rate to the different treatment modalities is 53% [13]. 

This report highlights the need for a high index of suspicion for breast cancer metastasis when such patients present with persistent GI symptoms that failed medical treatment. Although more common causes should be excluded, metastasis to the GI tract should be included in the differential diagnosis which may result in a significant alteration in the management and the outcome of these patients.
